# Soft-tissue and half-value windows outperform bone window in ureteral stone size measurements in non-enhanced computed tomography

**DOI:** 10.1177/02841851251406451

**Published:** 2025-12-23

**Authors:** Klara Sahlén, Anders Magnusson, Ulf Nyman, Marcin Popiolek, Lisa Wernroth, Mats Lidén, Johan Jendeberg

**Affiliations:** 1 Department of Surgical Sciences, Radiology, 8097 Uppsala University, Uppsala, Sweden; 2 Department of Translational Medicine, Division of Medical Radiology, Skåne University Hospital, Malmö, Sweden; 3 Department of Urology, 59566 Örebro University Hospital, Örebro, Sweden; 4 Department of Medical Sciences, Molecular Epidemiology, Uppsala University, Uppsala, Sweden; 5 Department of Radiology, Faculty of Medicine and Health, Örebro University Hospital, Örebro, Sweden

**Keywords:** Urinary tract, computed tomography, ureters, adults, calcifications/calculi, observer performance

## Abstract

**Background:**

Interreader variability in ureteral stone size measurements affect the predicted probability of spontaneous stone passage (SSP), especially in proximal ureteral stones. Window settings have been shown to influence interreader variability.

**Purpose:**

To investigate interreader variability of ureteral stone size measurements in four different window settings.

**Material and Methods:**

Patients with a unilateral proximal ureteral stone ≥2.0 mm detected during emergency computed tomography (CT) were included in this single-center study. Five observers measured each stone in three dimensions in a soft-tissue window, bone window, and two half-value windows (based on the mean [half-value MEAN] or maximum attenuation of the stone [half-value MAX]). Limits of agreement of the mean (LOAM) for stone size in each window setting were assessed. Logistic regression curves were created for predicted probability of SSP.

**Results:**

In total, 124 patients (87 men, 37 women; mean age = 52 years; age range = 22–82 years) were retrospectively evaluated. LOAM: bone window (±1.6 mm, 95% confidence interval [CI]=1.24–4.90), soft-tissue window (±0.4 mm, 95% CI=0.37–0.82), half-value MEAN window (±0.3 mm, 95% CI=0.24–0.40), half-value MAX window (±0.2 mm, 95% CI=0.14–0.30). Prediction curves aligned and shifted to the left as mean stone size decreased in the half-value window settings.

**Conclusion:**

The bone window is unsatisfactory for ureteral stone size measurements. The interreader variability in soft-tissue and half-value windows is on a sub-mm magnitude, with no expected impact on clinical decision-making. The half-value MAX window had the smallest interreader variability and should be considered for reproducible and semiautomated ureteral stone size measurements.

## Introduction

In acute ureterolithiasis, the chance of spontaneous stone passage influences the clinical decision between active stone removal and conservative treatment (1,2) Stone size and location in the ureter are known significant predictors of spontaneous stone passage (3–5). However, there is no international standardized method for stone size measurements in non-contrast-enhanced computed tomography (NCCT). This implies that the reported stone size can vary between readers and, from a research perspective, cause methodological inconsistencies between studies (6,7).

Stone size measurements are affected by how the retrieved data from the CT scan is processed and by the settings chosen in the picture archiving and communicating system (PACS), such as reconstruction plane, slice thickness, window setting, zoom level, and reported dimension of the stone (such as width or length). In two previous studies, a standardized measurement method was used and logistic regression curves created for spontaneous stone passage based on ureteral stone size and location (6,8). However, the interreader variability in bone window and soft-tissue window settings resulted in varied predictions of spontaneous stone passage, with the greatest impact on prediction for spontaneous passage of proximal ureteral stones.

**Fig. 1.**
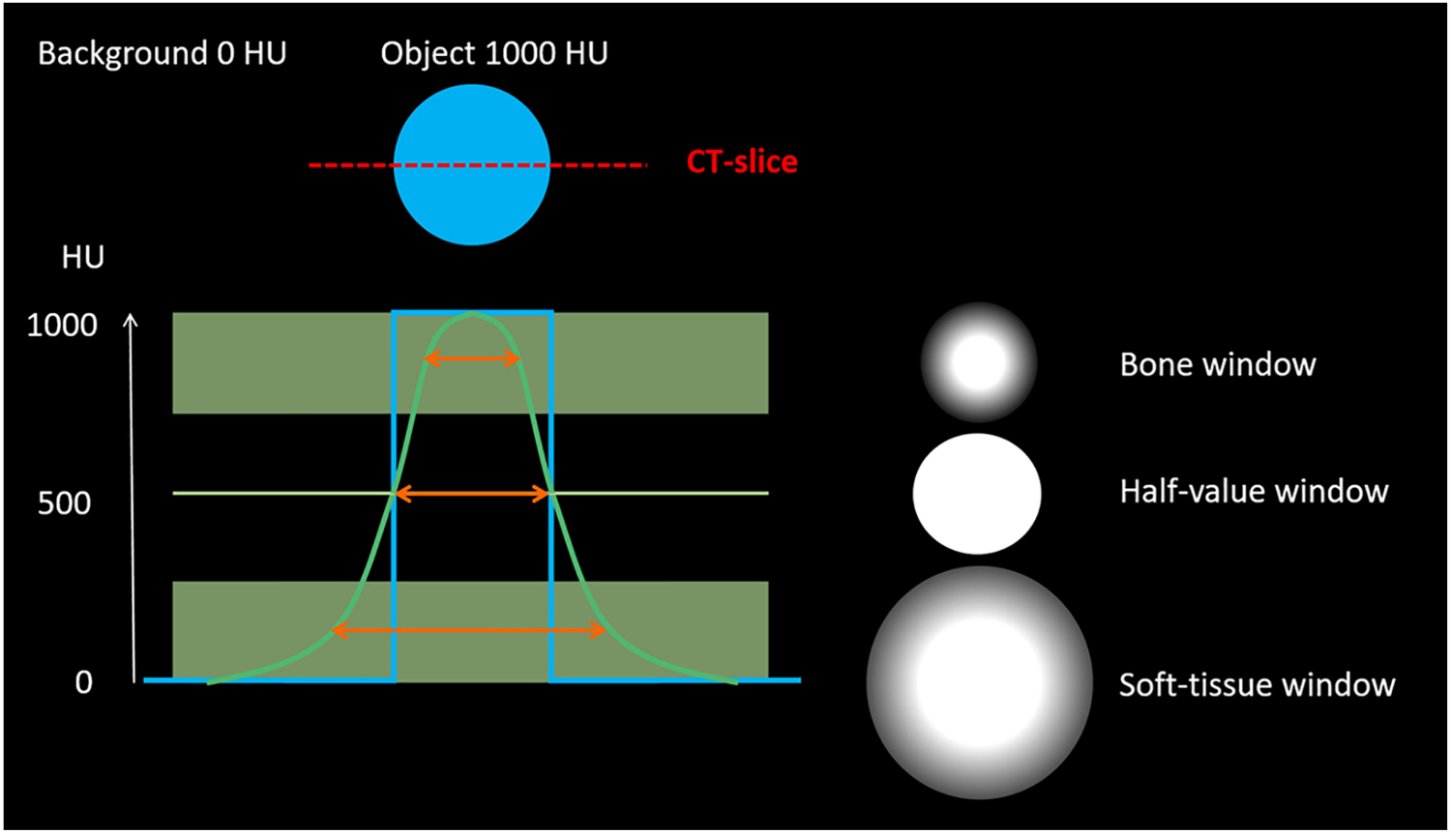
Theory behind the half-value window settings. The blue line in the graph represents the ideal attenuation curve of a dense object of 1000 HU. The green line represents the expected attenuation curve that will be obtained from the computer. If a measurement is performed within the upper green box (representing the bone window), the object will have an estimated size smaller than its true size. Conversely, measuring within the lower green box (representing the soft-tissue window) the object would be estimated to a larger size due to blooming artifact. Mathematically, the true size estimate of the object would be obtained at a window level set to half the value between the background attenuation and the maximum attenuation of the object when window width is set to zero, since the blooming artifact would then be eliminated.

Studies performed in the 1980s on the impact of window settings on the size and outline of a dense object in a CT image found that the most correct size of a dense object was obtained by adjusting the window center to half the value of the difference in attenuation between the object and the background (Fig. 1) (9). The demarcation of an object was found to become sharper with a narrow window width (10). Since ureteral stones are dense structures compared to their surroundings, this theory may be applicable for stone size measurements to reduce interreader variability.

In this study, two half-value window settings are introduced, referring to the half-value of the attenuation of the stone in Hounsfield units (HU). To our knowledge, no such window setting has been evaluated for the measurement of ureteral stones. Theoretically the true stone size and reproducible measurements could be obtained from a half-value window setting. The aim of the present study was to investigate the interreader variability in proximal ureteral stone measurements in four different window settings—bone window, soft-tissue window, and two half-value window settings—and to illustrate the impact of the interreader variability on predicted probability of spontaneous stone passage.

## Material and Methods

### Study population

The study was approved by the Regional Research Ethics Board (Dnr 2014/136) and the requirement for informed consent was waived.

The study retrospectively and consecutively included 124 patients (87 men, 37 women; mean age = 52 years; age range = 22–82 years) with a single proximal ureteral stone (located above the sacroiliac joint) ≥2.0 mm, (measured in the axial plane in a soft-tissue window setting, window center [WC] 50 HU, window width [WW] 400 HU) detected at an emergency NCCT at Örebro University Hospital, Sweden (April 2012 to September 2014).

**Fig. 2.**
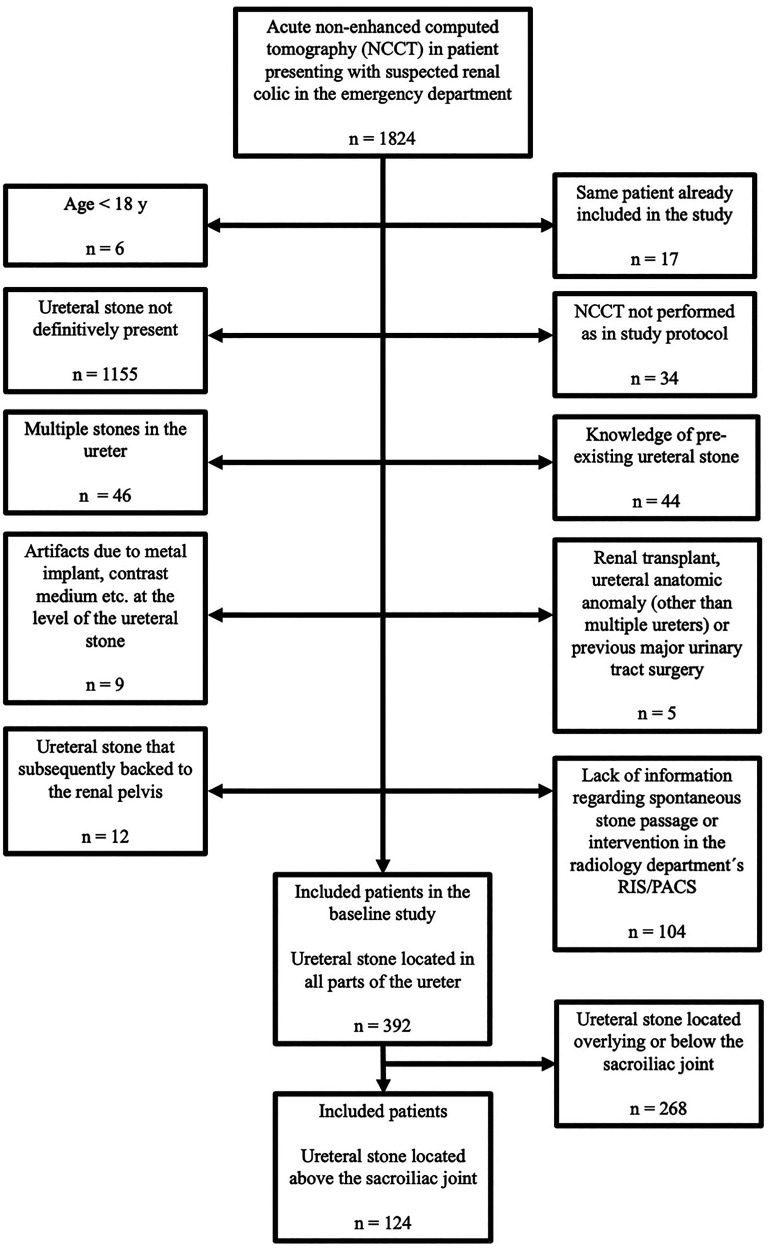
Exclusion criteria flow chart. The initial study included 392 ureteral stones. In this study, the population consisted of stones located in the proximal part of the ureter (n = 124; defined as located above the sacroiliac joint) and excluded the stones in the mid- and distal part of the ureter (n = 268; defined as located overlying or below the sacroiliac joint).

The study population is a subgroup of a previous study by Jendeberg et al. (8), in which the predicted probability of spontaneous stone passage based on stone size and location was demonstrated. The baseline study included 392 patients presenting with an acute single ureteral stone (located anywhere in the ureter) ≥2.0 mm. The stone size limit was chosen to reduce the risk of measuring something other than a ureteral stone and because ureteral stones <2.0 mm have a very high rate of spontaneous stone passage. Patients with a stone located in the upper part of the ureter (n = 124) were included in this study. This subgroup was chosen since the impact of stone size measurements on predicted probability of spontaneous stone passage has been found to be largest in patients with proximal stones (6). Exclusion criteria for the baseline study and this study are presented in Fig. 2.

### CT protocol

The CT examinations were performed on two different CT scanners. A total of 55 patients were examined using a 40-detector row CT scanner (Brilliance; Philips Medical Systems, Best, The Netherlands) with a low-dose NCCT protocol for the urinary tract with the following parameters: reference tube potential/effective tube loading = 120 kVp/70 mAs; volume pitch-corrected CT dose index (CTDIvol) = 4.9 mGy; collimation = 40 × 0.625 mm; standard filter (B); and supine position. In total, 69 patients were examined with a 2 × 128-channel scanner (Somatom Definition Flash; Siemens, Erlangen, Germany) with the following parameters: reference tube potential/effective tube loading = 120 kVp/70 mAs; CTDIvol = 4.7 mGy; collimation = 128 × 0.6 mm; filter B20f, B25f, or I30f; and supine position. Axial, coronal, and sagittal multiplanar reformations (MPR) of 3 mm were generated.

### Ureteral stone size measurements

The ureteral stones were measured using the PACS measurement calipers (Sectra IDS7, Linköping, Sweden) independently by five observers: three senior radiologists (UN, AM, JJ), one senior urologist (MP), and one radiology resident (KS). There was no consensus training in how to measure the stone before the measurements. The study material was pseudonymized and the observers were blinded to the initial CT report (except the inclusion criteria of the stone being ≥2.0 mm in the axial plane in a standardized soft-tissue window). Each stone was measured in 3 mm MPRs (axial, sagittal, and coronal planes), at a zoom level of at least 8×, in four different window settings (bone window, soft-tissue window, and two half-value window settings) defined in the next section. The sequences of window settings and cases were not randomized.

The largest stone diameter of the three measurements in the reformations was included in the statistical analyses. To avoid bias, at least 1 week had to pass between the measurements in each window setting and the results were registered in separate forms.

### Window settings

#### Soft-tissue and bone window settings

**Fig. 3.**
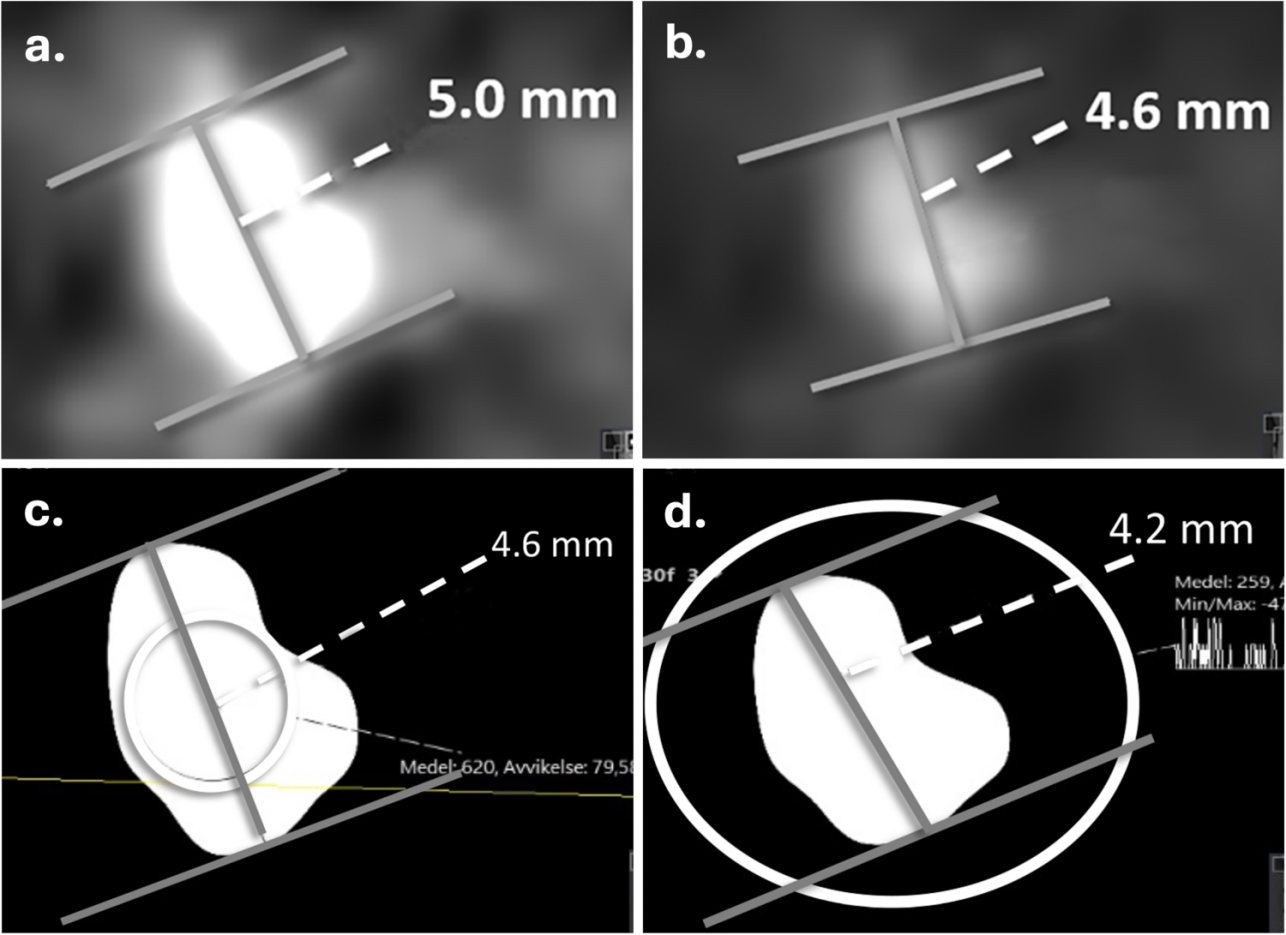
Soft-tissue and bone window settings. The same stone measured in (a) soft-tissue window: C 50 HU and W 400 HU; (b) bone window: C 300 HU and W1120 HU; and (c) half-value MEAN and (d) half-value MAX for comparison. C, window center; W, window width.

Soft-tissue window was defined as WC 50 HU and WW 400 HU (Fig. 3a) and bone window as WC 300 HU and WW 1120 HU (8,11) (Fig. 3b). The half-value MEAN and MAX window settings are shown for comparison (Fig. 3c, d).

#### Half-value window settings

The background surrounding a ureteral stone usually consists of fat or soft tissue with low HU values. For simplicity, an approximation of 0 HU was predefined as the background attenuation in the half-value window settings. Each reader adjusted the window center to half the value of the maximum or mean attenuation of the stone obtained in each measurement. The window width was set to 0.

#### Mean half-value window setting (half-value MEAN)

**Fig. 4.**
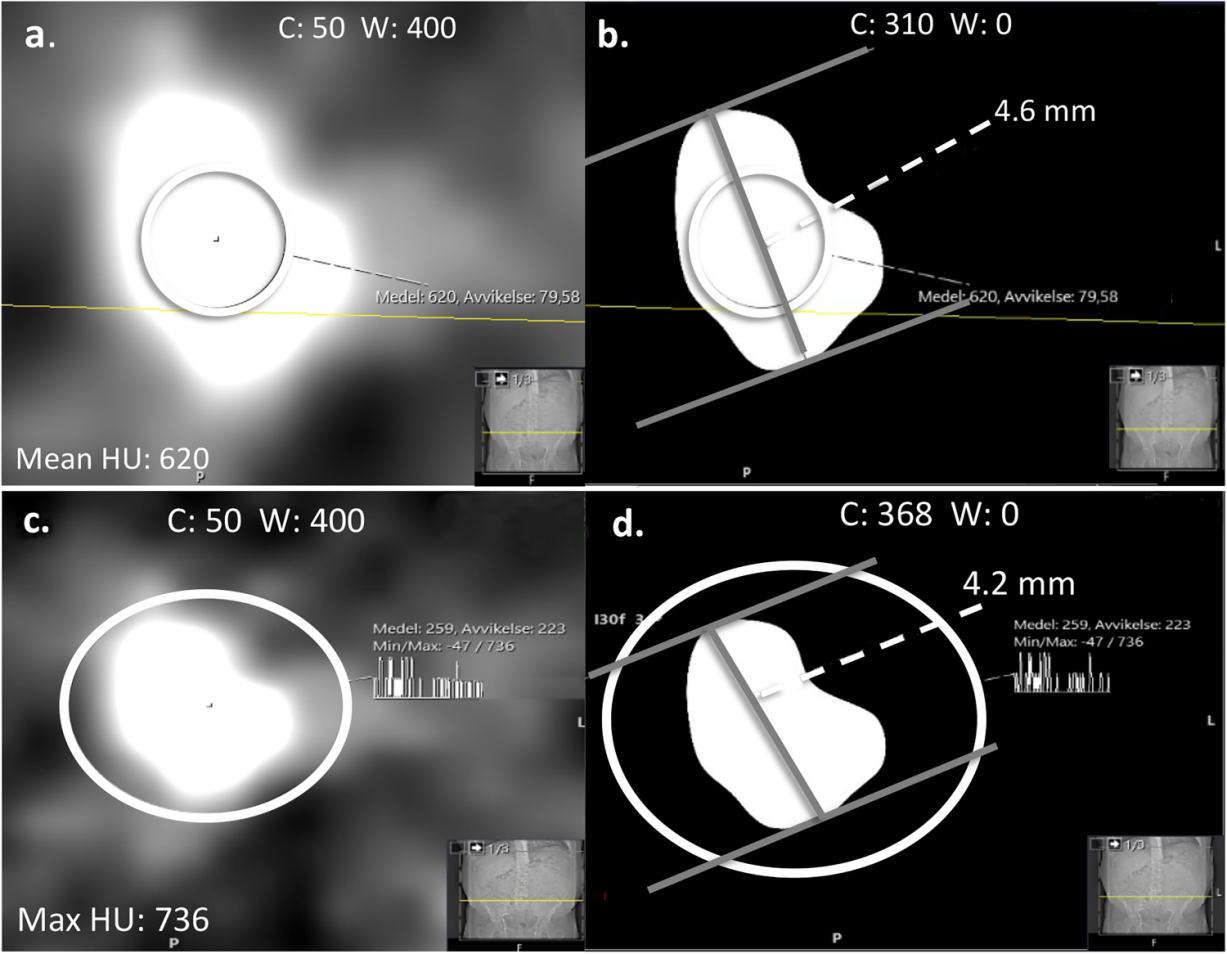
Half-value window settings. (a) Half-value MEAN was obtained from the mean attenuation value (620 HU) defined by the histogram circle, which included an area as large as possible within the borders of the stone but avoiding partial volume effect in the soft-tissue window, (b) C was set to half the mean attenuation value (310 HU) and W to zero. (c) Half-value MAX measurement was obtained from the maximum attenuation value (736 HU) defined by the histogram circle, which had to include the entire stone in the soft-tissue window setting, (d) C was set to half the maximum attenuation value (368 HU) and W set to zero. C, window center; W, window width.

In the half-value window setting based on the mean attenuation of the stone, the integrated histogram circle was placed in the center of the stone in the image where the stone was perceived as largest in the soft-tissue window. The histogram circle had to include an area as large as possible (within the borders of the stone to avoid partial volume effect) (Fig. 4a). WC was set to half the mean attenuation value of the stone and WW was set to 0 (Fig. 4b).

#### Maximum half-value window setting (half-value MAX)

To find the maximum half-value window setting based on the maximum attenuation value, the histogram circle was placed to fully include the stone in the image where the stone was perceived as largest in the soft-tissue window (Fig. 4c). WC was then set to half the maximum attenuation value of the stone and the WW was set to 0 (Fig. 4d).

### Spontaneous stone passage

Spontaneous stone passage was chosen as outcome since the primary reason for stone size measurements is to determine if the ureteral stone can be expected to pass spontaneously or requires intervention. Data regarding spontaneous stone passage was retrieved from the baseline study in which information of age, sex, and interventions were collected from the patient’s medical record. Spontaneous stone passage was defined as passage without intervention at a follow-up CT or intravenous urography, i.e. the patient had only received conservative treatment (watchful waiting, medical expulsion therapy) and/or analgesics. The follow-up examination performed closest to 140 days (20 weeks) after the emergency CT was used in this study.

### Statistical analyses

The largest stone size of the three reformation measurements for each observer was used in the statistical analyses. Agreement between the five observers was evaluated using Bland–Altman plots extended to multiple observers. The limits of agreement with the mean (LOAM) and associated 95% confidence intervals (CIs) were calculated using an additive two-way random effects model. The LOAM quantifies the extent to which an individual observer's measurement may deviate from the mean of all observers for a given subject and has been previously used in studies on agreement in CT measurements of small renal masses (12). To explore the predictive value of stone size, logistic regression models were fitted with spontaneous stone passage as the dependent variable and stone size as the independent variable. To visualize the relationship, the predicted probability of stone passage was plotted against stone size. All analyses were performed using R version 4.2.3 using the LOAMR (13) and the RMS (14). Due to a temporary technical error in retrieving the images in the PACS correctly for observer 5, the stone measurements in the bone window of this observer were excluded from the statistical analyses.

## Results

**Fig. 5.**
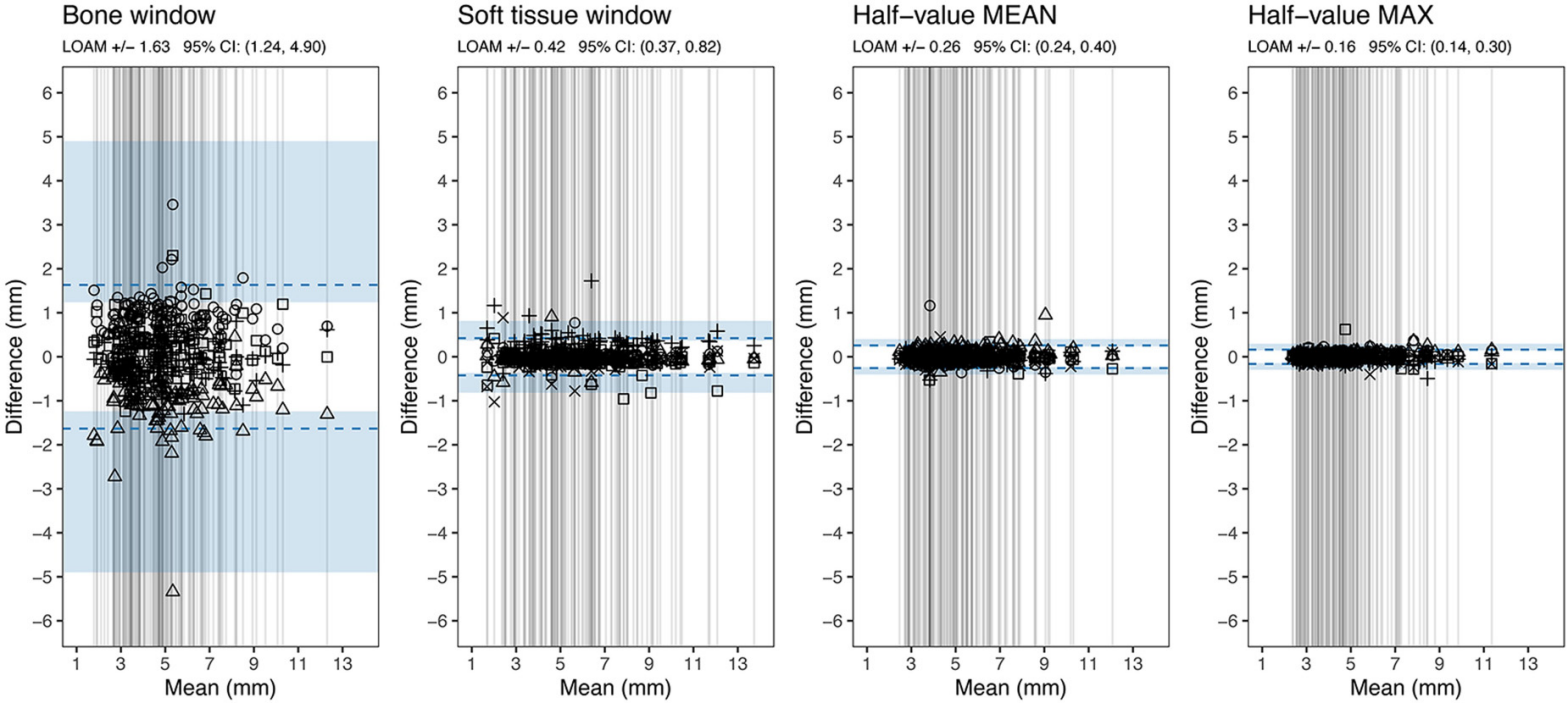
Bland–Altman plots showing the LOAM between the observers in different window settings. Each vertical line represents the mean value of all observers’ largest diameter for each stone (in mm) obtained in any of the reconstruction planes (axial, coronal, sagittal). The symbols represent an individual observer's measurement for that stone and are placed in the plot at the difference between the observer’s result and the mean value of all observers for that stone. The results of observer 5 were excluded from the analysis for the bone window setting. LOAM, limits of agreement.

In total. 124 patients (87 men, 37 women; mean age = 52 years; age range = 22–82 years) were included in the analyses. Spontaneous stone passage was observed in 68 (55%) patients, of whom 17 were women. Mean stone size ± standard deviation (SD) for all observers are shown in Table 1. Interreader variability in terms of LOAM using Bland–Altman plots are shown in Fig. 5. The bone window showed the largest variability (±1.6 mm, 95% CI = 1.24–4.90). This was evidenced by its non-overlapping 95% CI with the other window settings. In descending order, by the point estimate of variability the soft-tissue window followed (±0.4 mm, 95% CI = 0.37–0.82), then the half-value MEAN window (±0.3 mm, 95% CI = 0.24–0.40), and finally, the half-value MAX window setting (±0.2 mm, 95% CI = 0.14–0.30). However, among these three lower variability settings, only the soft-tissue window and the half-value MAX window had non-overlapping 95% CI.

**Table 1.** Stone size measurements.

Observer	Bone window	Soft-tissue window	Half-value MEAN	Half-value MAX
1	5.2 ± 2.0	5.6 ± 2.4	5.2 ± 1.8	4.8 ± 1.7
2	5.9 ± 1.9	5.7 ± 2.4	5.1 ± 1.8	4.8 ± 1.8
3	4.1 ± 2.0	5.6 ± 2.4	5.3 ± 1.9	4.8 ± 1.8
4	4.7 ± 2.0	5.9 ± 2.4	5.2 ± 1.8	4.7 ± 1.7
5	N/A	5.6 ± 2.4	5.1 ± 1.9	4.7 ± 1.7
All	5.0 ± 2.1	5.7 ± 2.4	5.2 ± 1.8	4.7 ± 1.7

Values are given as mean ± SD. The results of observer 5 in the bone window were excluded from the analysis due to a technical error.

N/A, not available; SD, standard deviation.

**Fig. 6.**
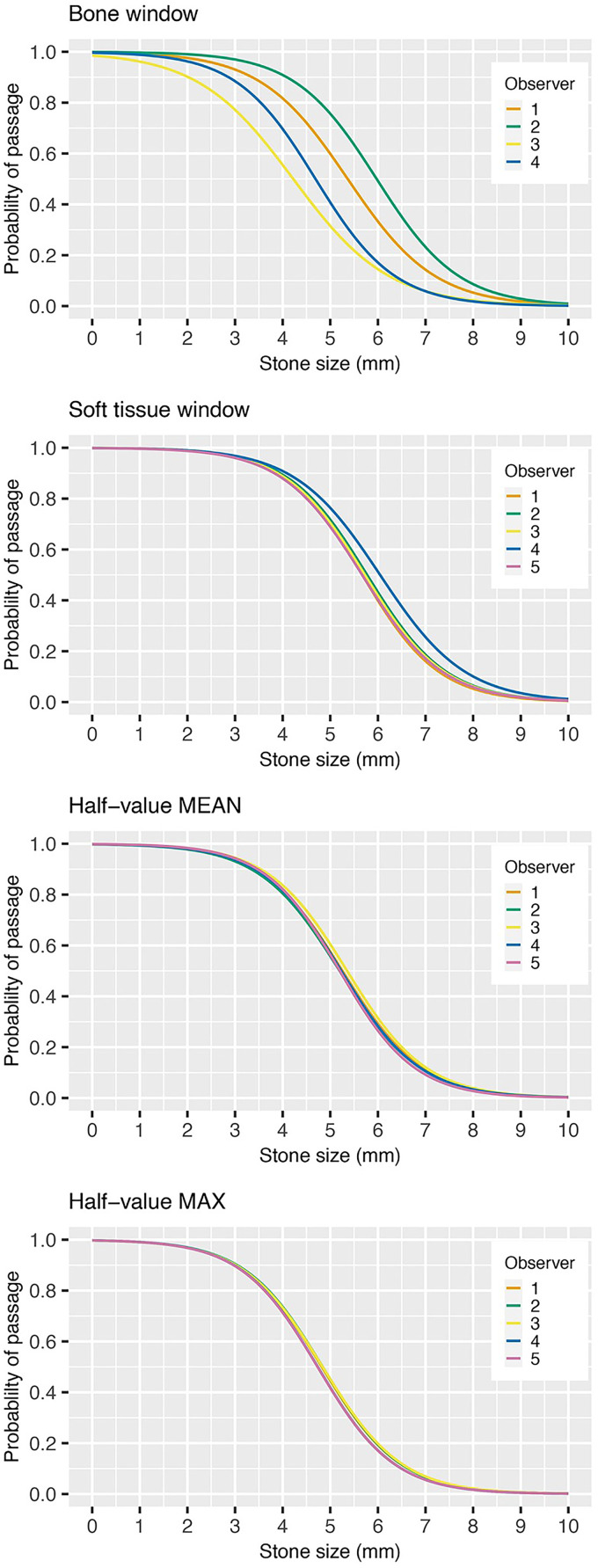
Probability of spontaneous stone passage as a function of stone size (mm) in four different window settings. The result of each observer is represented by colored lines reflecting interreader variability. The results for observer 5 were excluded in the bone window setting.

Logistic regression curves for probability of spontaneous stone passage as a function of largest stone size of the three measurements are shown in Fig. 6, for all observers in each window setting. The predictive curves (four in the bone window, five in the other window settings) are more spread out in the bone window and become more aligned in the soft-tissue and half-value windows. The curves are shifted towards the left in the graph as they align, corresponding to smaller measured stone sizes.

## Discussion

This study examined the interreader variability of ureteral stone size measurements in four different window settings including two half-value window settings, which to our knowledge have not previously been studied for ureteral stone size measurements. Interreader variability in ureteral stone size measurements can partly be explained by the blooming artifact associated with dense structures, which cause the object to appear larger than it is (15). By using a half-value window setting, the issue of blooming artifact may be reduced if not eliminated. A standardized measurement method that minimizes uncertainty of interreader variability is of interest because such a variability in measurements can influence clinical decision-making and reduce generalizability in research. The impact of interreader variability on predicted spontaneous stone passage is important since ureteral stone size is the most significant predictor of spontaneous passage. In the present study, interreader variability in the bone window setting was ±1.6 mm for four observers, in the soft-tissue window ±0.4 mm, half-value MEAN window ±0.3 mm, and smallest in the half-value MAX window setting of ±0.2 mm for five observers. The impact of the interreader variability is demonstrated in the regression curves for prediction of spontaneous stone passage (Fig. 6), by for example examining the measured stone size for each reader at 90% expected rate of spontaneous stone passage. In the bone window, the measured stone sizes were in the range of 2.0–4.0 mm between observers and in the soft-tissue window approximately 3.8–4.2 mm. The measured stone sizes were the same for all observers in both half-value windows: 3.5 mm in half-value MEAN and 3.0 mm in half-value MAX. The curves align and shift to the left as estimated stone size decrease. This implies that the expected predicted rate of spontaneous stone passage decreases for all stone sizes, apart from the smallest and largest stones. The mean stone size was largest in the soft-tissue window (5.7 mm), smaller in the half-value MEAN (5.2 mm) followed by bone window (5.0 mm) and half-value MAX (4.7 mm).

It is expected that the largest stone size is in the soft-tissue window since blooming artifact makes the stone appear larger. As the blooming artifact is minimized in the half-value windows, the size is affected by the level of the window center. In the half-value MEAN window setting, the window center will be lower compared to half-value MAX, resulting in a larger mean stone size (5.2 mm) compared to the half-value MAX measurements (4.7 mm). The smallest mean stone size is to be expected using the bone window, as explained in Fig. 1. However, in this material, half-value MAX had an even smaller mean stone size than bone window. This might be explained considering upper and lower gray levels: attenuation values become white over the upper level (WC + (WW/2)) and black below the lower level (WC − (WW/2)). In the bone window, the upper level is fixed at 860 HU (300 + (1120/2)) and lower level at −260 HU (300 − (1120/2)). The mean maximum attenuation of the stones in this material was 857 HU, which equals both the upper and lower gray level in the half-value MAX window setting since window width is zero. The less well-defined border of the stone in the bone window may cause interreader variability, which may result in larger stone size estimations. In previous studies, the bone window has been suggested as the best window for estimating the true size of the stone (11,16). We consider reproducibility of a measurement to be more important than finding the exact stone size. According to the results of this study, interreader variability will cause uncertainty up to several millimeters between readers in the bone window setting. The soft-tissue window setting is frequently used as the first window setting when examining a CT examination of the abdomen or urinary tract. For practical reasons, it is a suitable window for ureteral stone size measurements. This study found a small interreader variability (±0.4 mm), which may not be clinically significant. To obtain a half-value window, the measurement includes extra steps. This may not be tempting to a busy radiologist in daily work. However, the small interreader variability in half-value MAX is well suited for a semi-automated model to measure ureteral stone size. In practice, this would be useful but require a software developer to create such an algorithm in PACS and the algorithm to be validated. The utility of the maximum attenuation measurement is also already acknowledged, for example in nomograms to predict the success rate of shockwave lithotripsy in proximal ureteral stones (17,18) as well as to classify uric acid stones in a single energy CT (19). The overall interreader variability in the half-value windows was very small but not zero. As the half-values were set by the reader independently there is room for small variation. In what image the stone is perceived as largest and how the histogram is placed are also sources of interreader variability. The latter issue is limited by using the maximum attenuation. However, the issue regarding in what image the stone is measured is the same for all window settings.

The present study has some limitations. The retrospective nature of the study implies that follow-up and intervention were not standardized, and clinical management would affect the observation of spontaneous stone passage. Due to a technical error, we also had to exclude the measurements of one observer for the bone window, resulting in one less observer for the results in the bone window. Further, the patients were scanned in two separate CT machines and a comparison for inter-scanner variability was not performed in this study.

In conclusion, interreader variability in measurements of proximal ureteral stones is unsatisfactory using the bone window. In soft-tissue and the half-value windows, the interreader variability is on a sub-mm magnitude, with no expected impact on clinical decision-making. The smallest interreader variability was obtained using the half-value MAX window, which may be considered for reproducibility in research and for semi-automated measurements.
